# UT-1 Transporter Expression in the Spiny Dogfish (*Squalus acanthias*): UT-1 Protein Shows a Different Localization in Comparison to That of Other Sharks

**DOI:** 10.3390/biom14091151

**Published:** 2024-09-12

**Authors:** Christopher P. Cutler, Esosa Omoregie, Tolulope Ojo

**Affiliations:** 1 Department of Biology, Georgia Southern University, Statesboro, GA 30460-8042, USA; 2 Department of Biology, Baylor University, Waco, TX 76706, USA

**Keywords:** elasmobranchs, sharks, kidney, osmoregulation, salinity acclimation, urea transport, UT-1

## Abstract

The original UT-1 transporter gene was initially identified in the spiny dogfish (*Squalus acanthias*), but localization of the UT-1 protein was not determined. Subsequent UT-1 expression was shown to localize to the collecting tubule (CT) of the shark nephron in other shark species, with expression in a closely related chimaera species also located additionally at a lower level in the intermediate-I segment (IS-I) of the nephron. In spiny dogfish, two UT-1 splice variants are known (UT-1 long and short), and there was also a second UT-1 gene described (here termed Brain UT). In this study, a second splice variant of the second Brain UT gene was discovered. Expression profiles (mRNA) of UT-1 long and short and Brain UT were determined in a number of spiny dogfish tissues. Quantitative PCR in kidney samples showed that the level of the short variant of UT-1 was around 100 times higher than the long variant, which was itself expressed around 10 times higher than Brain UT cDNA/mRNA (in kidney). For the long variant, there was a significantly higher level of mRNA abundance in fish acclimatized to 75% seawater. Ultimately, three UT-1 antibodies were made that could bind to both the UT-1 short and long variant proteins. The first two of these showed bands of appropriate sizes on Western blots of around 52.5 and 46 kDa. The second antibody had some additional lower molecular weight bands. The third antibody was mainly bound to the 46 kDa band with faint 52.5 kDa staining. Both the 52.5 and 46 kDa bands were absent when the antibodies were pre-blocked with the peptide antigens used to make them. Across the three antibodies, there were many similarities in localization but differences in subcellular localization. Predominantly, antibody staining was greatest in the intermediate segment 1 (IS-I) and proximal (PIb) segments of the first sinus zone loop of the nephron, with reasonably strong expression also found at the start and middle of the late distal tubule (LDT; second sinus zone loop). While some expression in the collecting tubule (CT) could not be ruled out, the level of staining seemed to be low or non-existent in convoluted bundle zone nephron segments such as the CT. Hence, this suggests that spiny dogfish have a fundamentally different mode of urea absorption in comparison to that found in other shark species, potentially focused more on the nephron sinus zone loops than the CT.

## 1. Introduction

Marine elasmobranchs, such as the spiny dogfish (*Squalus acanthias*), are near osmoconformers with internal body fluids at a slightly higher osmolality than the environmental seawater [1,2]. However, they also tend to be ionoregulators, and while they have higher levels of body fluid Na and Cl ions than those found in teleost fish or mammals, the level is only around half that of seawater [2]. This higher level of body fluid osmolality is explained by the fact that they both keep and even make high levels of the nitrogenous waste product, urea, as well as utilizing significant amounts of trimethylamine oxide (TMAO; [2,3]). For this to work as an osmoregulatory strategy, they have to retain as much of these two molecules as possible. Although there are several potential routes for urea loss from the animals (gill, kidney, gastrointestinal tract/rectal gland, or skin), despite its relatively low urea permeability, the gill tends to be the biggest site of urea loss [4]. This is partly due to the fact that somewhere between 80 and 99% of urea in the glomerular filtrate is reabsorbed by the nephron [2].

The reabsorption of urea in the kidney necessitates the need for urea transporters, and the first of these (ShUT; hereafter referred to as dogfish UT-1 or just UT-1) was discovered in 1999 by Smith and Wright [5]. They showed UT-1 was expressed in kidney and brain and exhibited 2 mRNA sizes (4.3 and 2.2 kb, [5]). The transcript expressed in Xenopus oocytes exhibited phloretin-sensitive urea absorption into the oocytes [5]. Homologues of the UT-1 gene have since been identified in several other elasmobranchs (Atlantic stingray [6,7], houndshark [8], and bullshark [9]) as well as from the closely related holocephalon/chimaera elephant fish [10]. Indeed, in elephant fish, three versions of UT genes were identified (efUT1-3), along with 5′ end splice variant versions of efUT1 and 2 as well as the other gene [10]. Somewhat similarly, in the elasmobranch Atlantic stingray (*Dasyatis sabina*), 2 UT splice variant versions have been identified, but these produce a short and long version of the UT protein, with the long version having an extended C-terminal amino acid sequence [7]. In 2016, a transcriptomics study was published for the spiny dogfish [11], which, similar to the stingray, showed short and long versions of the UT-1 gene also existed in dogfish. Additionally, a partial second UT sequence was identified from dogfish brain cDNA (here called Brain UT). A dendrogram in the publication indicated that Brain UT was intermediate in similarity between UT-1 and UT-2/3 of the elephant fish, whereas UT-1 sequences (including dogfish UT-1) grouped together [11].

Elasmobranchs have a complicated renal nephron structure. This study of nephron segment terminology uses that of Cutler et al. [12], which is an amalgam of those of Hentschel et al. and Kakumura et al. [13,14], where the kidney has two zones, the sinus zone, where the nephron has two loops surrounded by blood sinuses, and the bundle zones with another 2 loops as well as the collecting tubule, all surrounded by a peritubular sheath [15]. After the glomerulus, the nephron segments are the neck segment (NS) and proximal Ia (PIa; first bundle zone loop), the proximal Ib (PIb), proximal II (PII), intermediate segment I (IS-I; all parts of the first sinus zone loop), intermediate segment II (IS-II), and early distal tubule (EDT; both second bundle zone loop); then the late distal tubule (LDT; second sinus zone loop); and the collecting tubule (CT; bundle zone) and collecting duct (CD; see [12] for model diagram). Additionally, the tubules in the bundle zone have a straight portion starting in the center of the sinus zone and extending across the kidney towards the lateral sides, followed by a convoluted lateral bundle zone, where the tubules run in a posterior–anterior direction with loop segments returning in the opposite direction. This is such that when the transverse kidney sections are seen, the straight bundle zone tubules are longitudinal, whereas the lateral convoluted bundle zone tubules are seen generally in cross-section, with classically, five tubule cross-sections in a ‘bundle’, namely, NS, PIa, IS-II, EDT, and CT, the EDT being recognizable by its larger sized diameter. Also, as discussed in Cutler et al. [12], cilia are present in abundance in the proximal half of the nephron (up to IS-II) but are absent from the LDT [16,17,18]. This difference was, therefore, used to distinguish between the similar sinus zone IS-I and LDT tubules using an anti-acetylated tubulin antibody, which stains cilia [12].

Some UT-1 in situ hybridization and immunohistochemical localization studies have been carried out in houndshark and bullshark, where UT-1 expression was found exclusively in the collecting tubule (CT) towards the end of the nephron [8,9]. In chimaera elephant fish, which has a similar nephron structure to elasmobranchs, in situ hybridization determined that UT-1 expression was found in the collecting tubule (CT) but also earlier in the nephron in the intermediate I (IS-I) tubule segment [14].

The original rationale for this study was to produce an antibody against the dogfish UT-1 amino acid sequence in order to have a marker to identify the collecting tubule (CT) segment of the nephron. But as is often the situation in research, the circumstances proved to be different and far more complicated than expected. UT-1 antibodies were made that would bind to both UT-1 variants (short and long), and ultimately three independent antibodies were made in order to better understand the results. Western blot analysis was performed to characterize the antibodies. The studies started with the availability in the gene bank of the dogfish UT-1 long and short nucleotide sequences as well as that of the partial Brain UT sequence. To further explore Brain UT, PCR amplification and sequencing of the 5′ end of the cDNA were carried out, and 3′ RACE was performed to complete the Brain UT cDNA coding region sequence. Finally, quantitative PCR (QPCR) was performed to identify any changes in mRNA expression in previously extracted and used kidney total RNA samples extracted from fish acclimated to different salinity environments [19,20].

## 2. Materials and Methods

### 2.1. Experimental Animal Samples

All animal samples used in this study were the same as those used for a number of previous studies (such as in [20]). As stated in those previous studies, IACUC approval of the Mount Desert Island Biological Laboratory and/or Georgia Southern University was obtained for all the animals used in this study. Details of exposure to different environmental salinities can be found in Cutler et al. [20,21]. Likewise, the same is true regarding the extraction of kidney total RNA samples.

### 2.2. DNA Cloning, Sequencing, and QPCR

Complementary DNAs for normal PCR reactions were produced as in Cutler et al. [21], with the exception that Superscript IV reverse transcriptase (Invitrogen/Thermofisher, Carlsbad, CA, USA) was used for cDNA synthesis reactions instead of Superscript III reverse transcriptase. Reactions were performed at 55 °C in a thermocycler and incubated for 30 min. cDNA samples were then diluted to 200 μL with deionized water, and 1 μL was used per 20 μL PCR reaction. Amplification of PCR fragments used Phusion DNA Polymerase (New England Biolabs, Ipswich, MA, USA) with 72 °C annealing and extension steps, and using supplied HF buffer, reactions were otherwise as in the manufacturer’s instructions. Primers were designed with a 72 °C annealing temperature using the manufacturer’s (New England Biolabs, Ipswich, MA, USA) online calculator. A similar batch version of the cDNA synthesis process was used for making cDNA for QPCR. Kidney total RNA samples had been previously normalized using their levels of 18S rRNA from several rounds of RNA agarose gel electrophoresis. Samples of each total RNA were diluted to 222 ng/μL of total RNA using a fixed volume of 10 μL of each RNA sample and varying the amount of dH_2_O added to create the correct concentration. This was performed to ensure the same level of pipetting error was associated with each RNA sample. The samples were mixed by vortexing and were kept on ice. A mastermix was then produced containing 1 μL of 100 μM Oligo (dT)_37_, 1 μL of 10 mM dNTPs, and 0.5 μL of SUPERase.In Then, a thermostable RNAse inhibitor (20 units/μL; Life Technologies, Carlsbad, CA, USA) was added per sample. This was mixed, and 2.5 μL was added to a set of labeled 200 μL PCR tubes held at 0 °C in a cool block. Then, 4.5 μL of each of the normalized and diluted kidney RNA samples was added to each PCR tube, and the contents were mixed. Samples were then transferred to a thermocycler and heated to 65 °C for 5 min, followed by cooling to 0 °C. PCR tubes were then centrifuged at 0 °C to collect the contents. Meanwhile, a second mastermix of 2 μL of Superscript IV RT buffer, 0.5 μL of 100 mM Dithiothreitol, and 0.5 μL of Superscript IV reverse transcriptase (200 U/μL) per sample was prepared, mixed, and added to each tube at 0 °C, and these were again mixed. The tubes were then heated to 45 °C for 30 s in a thermocycler to anneal the Oligo dT primer, and then the temperature was raised to 55 °C for 30 min. The temperature was then further raised to 80 °C for 10 min to inactivate the reverse transcriptase. Finally, the tubes were placed back in the cool block at 0 °C, and 190 μL per sample of dH_2_O was added to each one. The tubes were then mixed by vortexing and stored frozen at −80 °C until use.

The DNA cloning and sequencing was carried out on the RPL-P0 cDNA fragment, Brain UT cDNA 5′ end fragment, 3′ RACE fragment, and the QPCR fragments, which used a pCR4-TOPO TA cloning kit for sequencing; it was carried out in a similar fashion to the study [22] (see also Table 1). The 3′ RACE used brain cDNA made previously using a Takara Smarter RACE kit [22]. PCR fragments made with Phusion DNA polymerase were excised from agarose gels and purified using a Monarch DNA purification kit (New England Biolabs, Ipswich, MA, USA), followed by ‘A-tailing’ using 1.25 units of Taq DNA polymerase, 1× standard Taq Polymerase buffer (both New England Biolabs, Ipswich, MA, USA), and 0.2 mM dATP. PCR tubes with purified fragment DNA were then incubated at 72 °C for 10 min. This was necessary to facilitate the TA cloning of the otherwise blunt-ended DNA fragments. Colonies from cloning were picked off agar plates with sterile toothpicks and grown in terrific broth for >18 h overnight at 37 °C. Inserts were assessed using colony PCR amplifications (using Taq DNA polymerase; New England Biolabs, Ipswich, MA, USA) of the plasmid vector region containing inserts. PCR reactions with plasmids containing appropriate DNA inserts were purified using a Quickstep 2 PCR purification kit (Edge Biosystems, Gaitherburg, MD, USA) and quantified using agarose gel electrophoresis and logic DNA ladder (Lamda Biotech, St Louis, MO, USA). Twenty ng per 1 Kbp of DNA was then pipetted into a PCR tube together with 4 pmol. of either extralong (XL) T3 or T7 primers. Sequencing reactions were sent to Eton Biosciences (Durham, NC, USA) for Sanger DNA sequencing. The gene bank accession numbers for the new sequences from this study were as follows: Brain UT, PQ050617; Brain UT spliceoform, PQ047459; and RPL-P0, PQ047458.

For quantitative PCR, frosted 96-well plates were used (4titude) with microamp optically adhesive film covers (Applied Biosystems/Thermofisher, Foster City, CA, USA). For each QPCR amplification, test reactions were run at a series of annealing temperatures, often using a number of versions of primers to ensure that only a single product was amplifying in the reaction using melt curve analysis. Total RNA controls were run to assess for genomic DNA amplification. This was because the RNA samples used to make cDNAs were known to contain small amounts of genomic DNA contamination. At least one primer site of each primer pair was located across a putative exon–intron junction to prevent genomic amplification. Products produced were also cloned and sequenced once the primer pair to be used was determined. To create a scale to get copy number estimates from amplifications, QPCR fragments were amplified, purified, and using their molecular weight and Avogadro’s number, the number of DNA molecules per μL was calculated. A top standard of 100 million DNA copies (per 4.5 μL) was then created and diluted 1 in 10 down to 100 copies (per 4.5 μL). The standards were then run in duplicate alongside the gene fragment’s actual cDNA/QPCR reactions. This approach has several advantages over relative QPCR methods. Principally, it allows comparisons between gene copy numbers even when the efficiency of the QPCR reactions is different because the standard reactions are run identically to the cDNA reactions using identical parameters. For technical reasons, it does not give an absolutely perfect quantitative estimate of the number of mRNA molecules for a particular gene in the RNA samples. But errors in the estimate, such as the presence of a small amount of RT buffer in cDNA samples or the fact that reverse transcriptase can create more than one cDNA off a single mRNA, are the same for every cDNA/QPCR gene run. To allow flexibility in the analysis of the data, a housekeeping gene used previously in other species, RPL-P0, was cloned from *Squalus acanthias* cDNA and was also run on the same kidney cDNA samples, but this gene gave a significant difference between groups and so was not used. A GAPDH gene has also been run, but it did not improve the data. Overall, it is worth reiterating that the kidney RNA samples were already normalized at the start of experiments against 18S rRNA levels, so further use of a housekeeping gene was unnecessary and potentially counterproductive (as housekeeping genes can also change expression when fundamental changes are made to the circumstances of experimental animals, as was the case here with RPL-P0). 

For the QPCR samples (n = 6 fish/samples per group), a mastermix was made containing 5 μL 2xLuna Universal qPCR mastermix (New England Biolabs, Ipswich, MA, USA) and 0.25 μL of each 10 μM primer and 5.5 μL added to wells of the plate at 0 °C, and then triplicates of 4.5 μL of each diluted cDNA or duplicates of standard DNA were added to wells. Plates were centrifuged at 2000 RPM in a Beckman (Indianapolis, IN, USA) J30i centrifuge using a JS5.9 swinging-bucket rotor before amplification. Reactions were run and analyzed on a QuantStudio6 Flex Real-time PCR Instrument (Applied Biosystems/Thermofisher, Foster City, CA, USA). Data were processed on a Microsoft Excel spreadsheet (version 16.16.27). ANOVA statistics with Fisher Post hoc testing was carried out using Statsview software (Abacus concepts, Mindvision software, Version 4.01, Adelaide, Australia). Gene alignments were performed using Genejockey II software (Biosoft, Version II, Cambridge, UK).

### 2.3. Antibodies, Western Blots, and Immunohistochemistry

**Figure 1:**
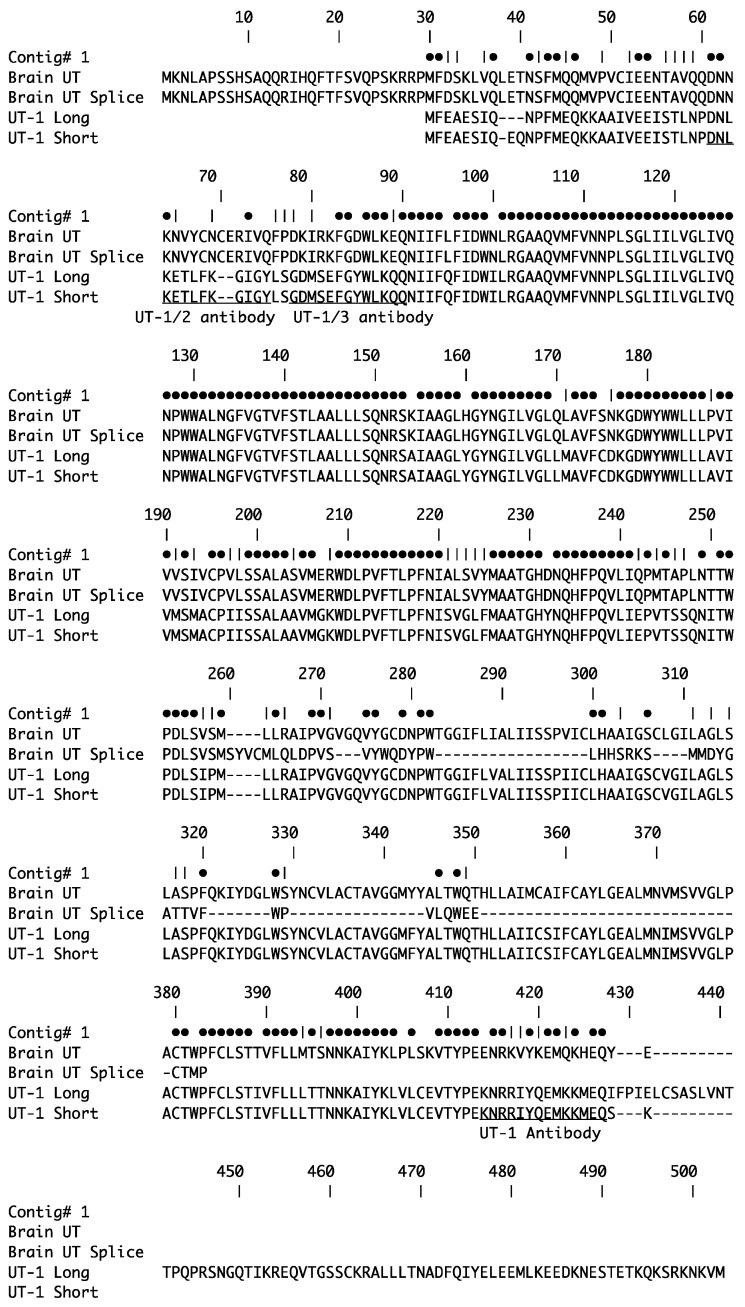
Amino acid alignment (single letter codes) of the complete sequences of *Squalus acanthias* Brain UT (Accession No. PQ050617) and its splice variant, Brain UT splice (Ac. No. PQ047459). Included for comparison are the UT-1 short (Accession No. AF257331 and HAGV01114644) and long (Accession No. HAGV01114645) sequences [5,11]. Dots above the alignment denote positions where the amino acids are the same. Vertical lines indicate where chemically similar amino acids are at that position. Underlining indicates the amino acid sequences used for peptide antigens for antibody production. There are between 4 and 8 amino acids in common between UT-1 and Brain UT in the antigenic regions.

UT-1 antibodies in this study were all rabbit custom-made affinity-purified polyclonal antibodies made by Genosphere (Paris, France). The company provides independent production and purification of antisera from two New Zealand white rabbits. Results from the pairs of rabbits have always been close to identical, with slight differences in titer. A mouse anti-acetylated tubulin monoclonal antibody (T6793; Sigma, St Louis, MO, USA) was used to label cilia present in the proximal part of the nephron [12]. The peptide antigens used to generate the UT antibodies were as follows: UT-1 NH2-CKNRRIYQEMKKMEQ-COOH, UT-1/2 NH2-PDNLKETLFKGIGYC-COOH, and UT-1/3 NH2-SGDMSEFGYWLKQQC-COOH (see Figure 1). For each peptide, a terminal cysteine was added to each sequence (as shown) to allow attachment to a keyhole limpet hemocyanin (KLH) carrier protein.

Western blotting was carried out essentially as presented in [19]. Moreover, crude membrane tissue extracts and purified plasma membrane derived from those samples were also used (see [20]). Peptide antigen blocking control blots were carried out by adding 50 μg/mL peptide in a 1 mL TNT buffer volume (i.e., 50 μg peptide) to the antibodies for >1 h before the blocked antibody was then added to the remaining buffer incubating the Western blot.

Immunohistochemistry used already available paraffin wax embedded kidney tissue blocks and was carried out as described in Cutler et al. [12], except that a Tyramide SuperBoost amplification kit (Thermofisher) was used for images involving the UT-1 antibody as well as some of the images for the UT-1/2 antibody, as indicated in figure legends (see also [20]).

## 3. Results

The complete coding region of Brain UT was cloned and sequenced (see Figure 1). The sequence was a completed version of the partial DNA sequence in the gene bank (Accession number HAGW01085140; [11]). The nucleotide homology of the Brain UT cDNA sequence compared to that of UT-1 short was 81%. Additionally, an apparent splice variant of Brain UT was isolated from brain cDNA. This was presumably missing an exon (or exons) of 100 bp in comparison to Brain UT itself. Because of the omission, the amino acid reading frame of the sequence was shifted after the missing piece, resulting in a different and shorter translated C-terminal amino acid sequence. This splice variant could not be amplified from kidney cDNA. The RPL-P0 control gene sequence was unavailable for the spiny dogfish, so primers were designed using a sequence alignment of RPL-P0 sequences from the cat shark (*Scyliorhinus canicula*; AY392168.1), the whale shark (*Rhincodon typus*; XM_020527693), and the elephant fish (*Callorhinchus mili*; JX207461) to identify conserved regions of nucleotide sequence. At the identified locations, the sequence found in the whale shark was used to make the RPL-P0 primers. The dogfish 775 nucleotide sequence obtained, shared 91% nucleotide homology with that of the cat shark and 90% with the whale shark. 

**Figure 2:**
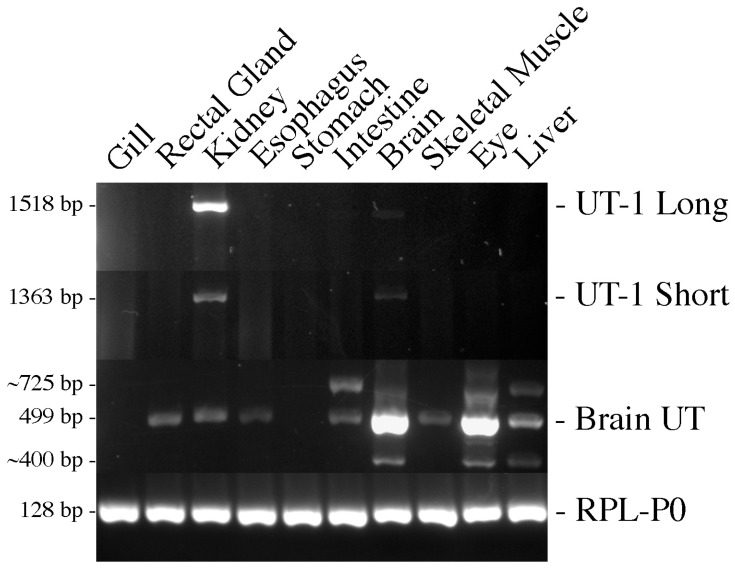
Agarose gel electrophoresis of RT-PCR reactions of UT-1 long, UT-1 short, Brain UT, and RPL-P0 using different tissue cDNAs from the dogfish. The Brain UT PCR amplifications resulted in multiple extra bands whose identity is unknown. The RPL-P0 amplifications used the QPCR primers. The UT-1 primers amplified up the full-length coding regions to check for any additional splice variants.

To determine where the various genes were expressed, RT-PCR amplifications of UT-1 long and short, Brain UT, and the control gene RPL-P0 were performed on various tissue cDNAs (see Figure 2). UT-1 long and short were both predominantly expressed in the kidney, and, to a lesser extent, in the brain, as previously reported on stringent Northern blots [5]. Brain UT mRNA/cDNA was highly expressed in the brain and eye but showed fairly widespread low levels of mRNA expression in most other tissues (except the gill and stomach), including the kidney. Three or four other bands were amplified along with the main Brain UT transcript. These are unrelated to the splice variant, also isolated separately, but what they represent is unknown.

**Figure 3:**
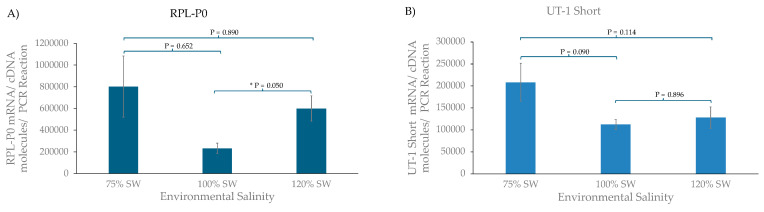

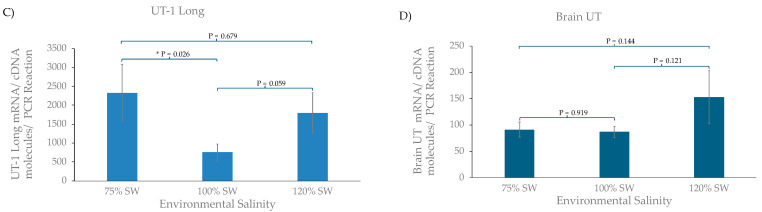
Quantitative PCR (QPCR) results using kidney cDNA for (**A**) RPL-P0 control gene, (**B**) UT-1 short, (**C**) UT-1 long, and (**D**) Brain UT. * = probability values (*p*) of less than 0.05.

The quantitative PCR for the RPL-P0 control gene showed a significant difference between fish acclimated to 100% SW and 120% SW, although the 75% SW-acclimated group was also elevated compared to the animals in the normal SW (100%) group (see Figure 3). This suggests that the animals are significantly stressed by the osmotic environment, and that is causing upregulation in the protein synthesis machinery (RPL-P0 is a ribosomal protein). Similar trends were seen with UT-1 long and short, but only the difference between the 75% and 100% SW animals for UT-1 long was statistically significant. The Brain UT results were somewhat different, with only the 120% SW group elevated a lot compared to the 100% group, but this was not a statistically significant increase. The other interesting feature of the results was the approximate number of transcripts per PCR reaction. The levels for UT-1 short were around 100× greater than that for UT-1 long, which were again around 10× the level for Brain UT. This may explain why, in the original study of isolating the dogfish UT-1 cDNA, only UT-1 short was discovered [5].

**Figure 4:**
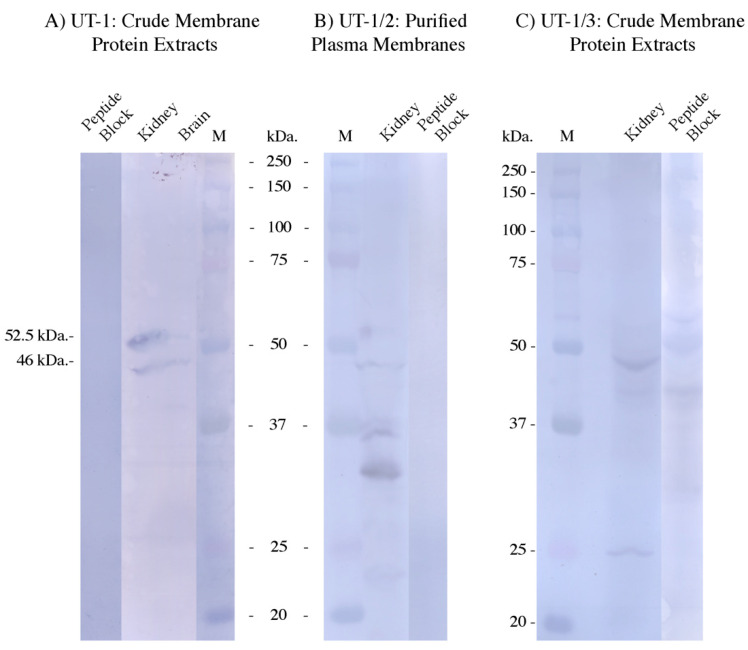
Western blots of *Squalus acanthias* protein crude extracts (400 μg; (**A**,**C**)) or purified plasma membranes (300 μg; (**B**)) from kidney and or brain tissue as indicated. Molecular Weight Protein marker (M) was Precision Plus Protein Kaleidoscope marker (Biorad, Santa Rosa, CA, USA). Peptide block refers to blots incubated with antibody pre-blocked (50 μg peptide/mL for >1 h) with the peptide antigen used to make the antibody. For the UT-1 antibody blot, the blot was one of four blot strips from one filter, with only one marker lane (M) which was located on a different strip, and so that was scanned separately and aligned with the UT-1 blot. All the peptide-blocked blot lanes were also cropped and slightly resized to align them next to each blot to aid comparison. Original scans are available on request.

The UT antibodies showed variable levels of signals in immunohistochemistry and Western blotting. The UT-1 antibody was the strongest, with UT-1/3 somewhat weak and UT-1/2 very weak. In the Western blotting (Figure 4), the expected size of UT-1 long was 51.6 kDa and UT-1 short 43.4 kDa. With the initial C-terminal antibody, UT-1, two bands of 52.5 and 46 kDa were obtained, which were very strong in kidney with lower levels also seen in the brain. There were also faint bands seen at around 40 kDa in the brain and around 25 kDa in the kidney. All the bands were absent in the peptide-antigen-blocked antibody control tracks. With the N-terminally located UT-1/2 antibody, no appreciable bands were seen using crude membrane protein extracts. So purified kidney plasma membrane protein was used instead, and this also gave bands of around 52.5 and 46 kDa, but a number of lower molecular weight bands were also present. All these bands were blocked when the antibody was incubated with the peptide-antigen (i.e., in the control). The UT-1/3 antibody (which was also N-terminally located) mainly gave a band of around 46 kDa, with very faint staining at 52.5 kDa. There were also faint bands around 42 kDa and 25kDa, while the 46 and 25 kDa bands and the faint 52.5 kDa staining were absent in the peptide-antigen-blocked antibody control. There was still a band around 42 kDa. Interestingly, some other bands also appeared. Due to the experience of performing blots for a number of years, it seems some peptide antigens, when used in the controls at relatively high concentrations, can themselves bind to either other proteins on the blot (attaching the antibodies and giving banding) or the PVDF filter (giving a more general background outside of the protein track, which is itself blocked out by the protein sample). This is variable from one peptide-antigen to another.

In the immunohistochemistry, overall, it was clear that while some nephron/tubule segments stained well with the three antibodies, with all three, there were some similar tubules with little or no staining. This was starkest with the very weak UT-1/2 antibody, with only a very few tubules showing staining across the whole kidney transverse cross-section. 

**Figure 5:**
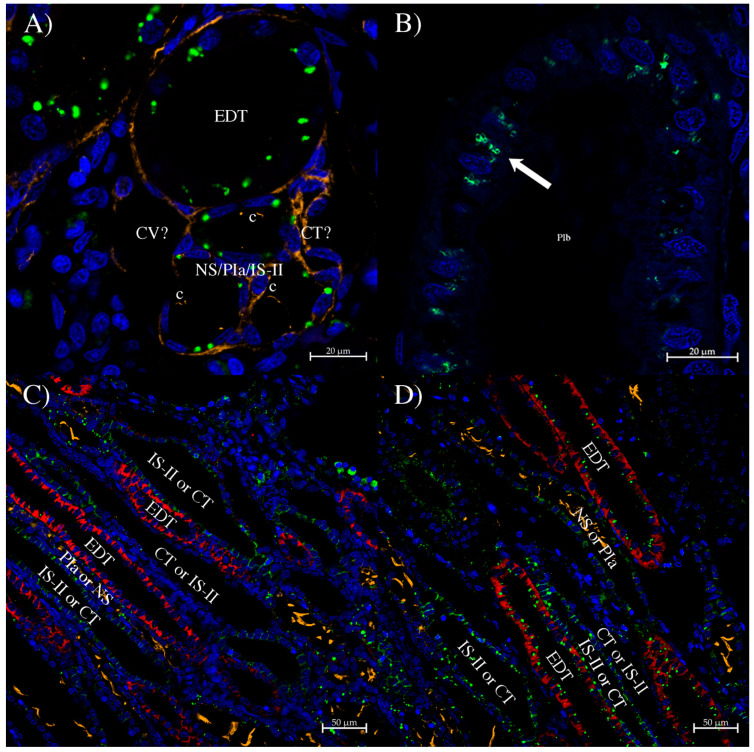
Immunohistochemistry using the UT-1 antibody. (**A**) Punctate cytoplasmic UT-1 staining seen in the convoluted bundle zone where the five tubules of the bundle are seen in cross-section. c = cilia staining. (**B**) Aryscan-enhanced resolution image of punctate staining of a sinus zone PIb tubule. (**C**,**D**) Examples of UT-1 staining in straight bundle zone tubules. On all sections, UT-1 antibody staining is green (Alexa 488 dye) using tyramide amplification, anti-acetylated tubulin antibody cilia stain is orange (Alexa 555 dye), Squalus AQP4/2 antibody staining the EDT (see [12]) is red (CF633 dye), and Dapi nuclear counterstain is blue.

**Figure 6:**
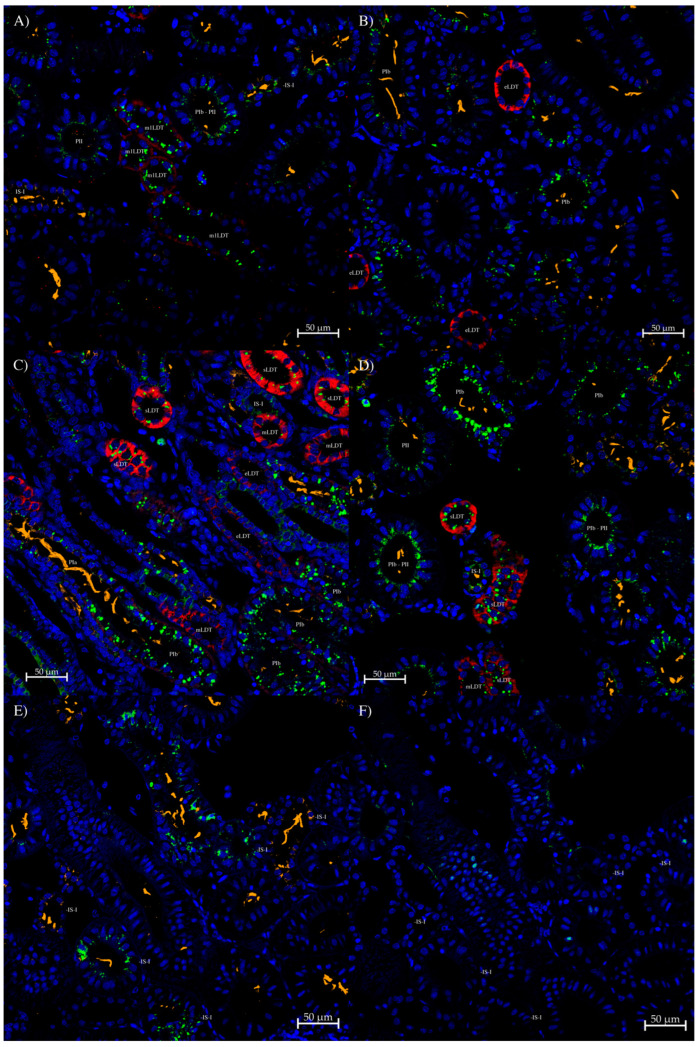
Immunohistochemistry using the UT-1 antibody in the sinus zone of the kidney. (**A**) Punctate cytoplasmic UT-1 staining seen in the IS-I, mid-LDT (mLDT; AQP3 antibody, red), and lower level staining in PII tubules. (**B**) Staining in the PIb tubule segment but no staining in tubules where AQP3 staining (red) is strong, at the end of the LDT (eLDT). (**C**) Strong UT-1 staining in the PIb tubule segment, with a tubule transitioning from the PIa of the straight bundle zone to PIb. Also strong staining at the start with less in the middle of the LDT tubules (sLDT and mLDT; with AQP4/2 staining, red). (**D**) Strongest punctate UT-1 staining was found in some PIb tubules. Staining localized to the sLDT and mLDT is also seen with AQP4/2 staining (red). (**E**) UT-1 staining in some but not all IS-I tubules. (**F**) Serial (to section (**E**)) control section incubated with peptide-antigen-blocked UT-1 antibody, which exhibits no similar staining (to that on section (**E**)). On the sections, UT-1 antibody staining is green (Alexa 488 dye) using tyramide amplification (in (**A**–**D**)), anti-acetylated tubulin antibody cilia stain is orange (Alexa 555 dye), Squalus AQP4/2 or AQP3 antibody staining (see [12]) is red (CF633 dye), and Dapi nuclear counterstain is blue.

The UT-1 antibody (Figure 5 and Figure 6) showed mostly punctate staining in the cell cytoplasm with a small amount of staining occasionally at the apical pole of the cell (e.g., PII, Figure 6A), although that could be a matter of its lower intensity. The punctate staining was shown in high-resolution Aryscan images to be in membranous cytoplasmic structures (Figure 5B), generally on the apical side of the nucleus and so presumably located in the endoplasmic reticulum or Golgi. Staining was most intense in the sinus zone PIb tubule segment (Figure 6D), which can be observed in a tubule emerging from the straight bundle zone PIa segment (Figure 6C). Almost as strong staining was seen in IS-I tubules and the start (sLDT) and middle (mLDT) of the LDT loop (Figure 6A,C–E). The end of the LDT loop, where AQP3 staining was strongest (eLDT), showed no staining (Figure 6B). Occasional lower level staining was seen in the PII segment, probably in places where the tubule was transitioning from PIb to PII segments. In convoluted bundle zone cross-sections (Figure 5A), sparse dots of punctate staining were seen, although, overall, the level was generally very low. The EDT segment showed consistent staining, but of the other four tubule segments (in the bundle), usually two, occasionally three, showed staining, and on one occasion, possibly all four other (non-EDT) tubule segments had punctate dots of staining (Figure 5A). Which of these tubules is which (neck segment [NS], proximal 1a [PIa], intermediate segment II [IS-II], collecting tubule [CT], or even the non-nephron central vessel, CV)), is hard to identify. Despite that, it is clear that the CT segment did not express much UT-1 protein, and it is possible that there was none, and there was no sign of membrane staining at all. In the straight bundle zone, there was patchy staining, with some punctate staining in some parts of the EDT (identified with AQP4/2 antibody; red). There was sometimes lower-level staining in more proximal tubule segments (which have cilia in them, i.e., NS or PIa; tubulin-staining orange). There were two other tubules, one unstained and one with both general cytoplasmic staining and punctate staining, which were distal tubules (due to lack of cilia) either IS-II or the CT.

**Figure 7:**
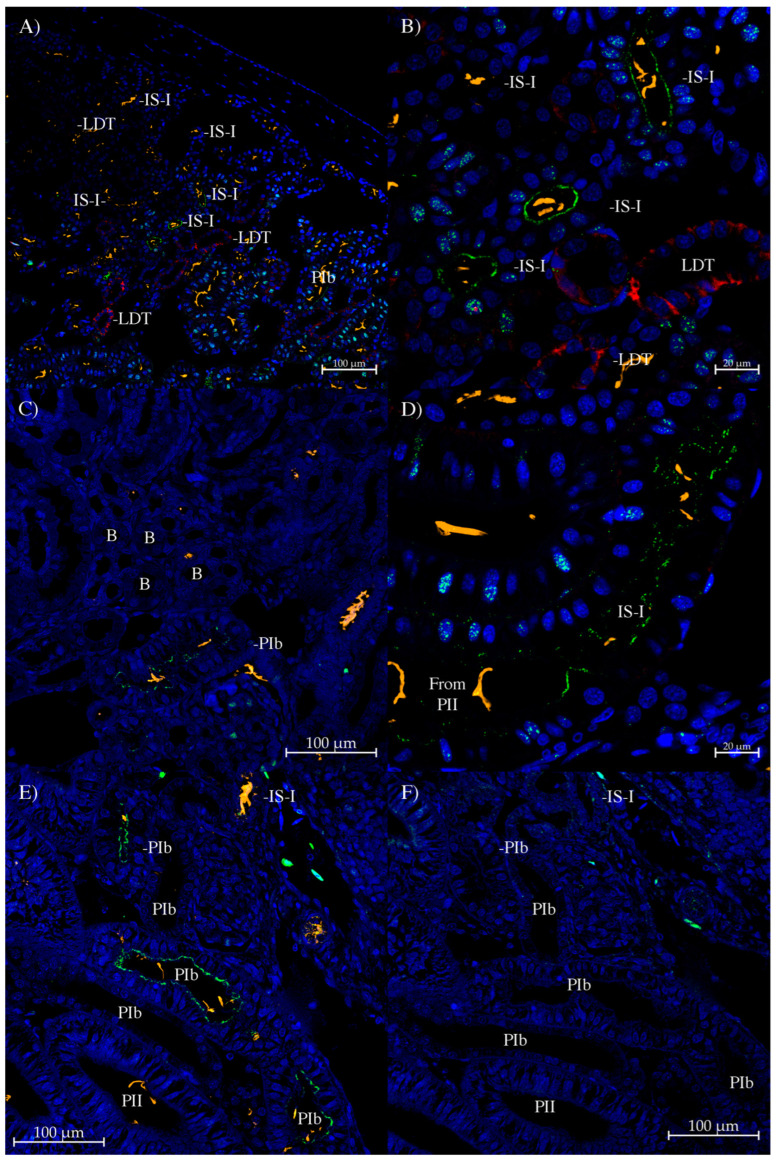
Immunohistochemistry using the UT-1/2 antibody. (**A**) A wide-field image of part of the kidney showing the weak apical membrane staining of some IS-I tubules and a PIb tubule. There was no apparent staining of the LDT tubules (red; AQP3 antibody). (**B**) High-magnification image of (**A**). (**C**) Shows a transitional region with both bundle (top left) and sinus (bottom right) zones. Bundle tubules in the bundle zone (marked with ‘B’ in the EDT tubule of each bundle) showed no staining. (**D**) Apparent transition between the PII part of a nephron into the IS-I, with a gradual increase in patchy staining. (**E**) A section with three PIb tubules showing apical staining. (**F**) A serial (to section (**E**)) control section incubated with peptide-antigen-blocked UT-1/2 antibody, which exhibits no similar staining (to that on section (**E**)). On the sections, UT-1/2 antibody staining is green (Alexa 488 dye; using tyramide amplification for sections (**D**–**F**)), anti-acetylated tubulin antibody cilia stain is orange (Alexa 555 dye), Squalus AQP3 antibody staining (see [12]) is red (CF633 dye), and Dapi nuclear counterstain is blue.

For the UT-1/2 antibody, staining was very weak, with staining only found in a few IS-I tubules initially (Figure 7A,B). With tyramide amplification, more staining was apparent, including PIb segments of the nephron (Figure 7E). No staining was seen in PII or LDT segments, possibly due to the weak binding of the antibody. Likewise, there was no apparent staining in the bundle zone tubule segments (Figure 7C). On IS-I tubule sections, the line of staining appeared to be at the apical membrane of the tubule cells. In the PIb segments, the line of staining appeared to potentially be just underneath the membrane, but that was not 100% clear.

**Figure 8:**
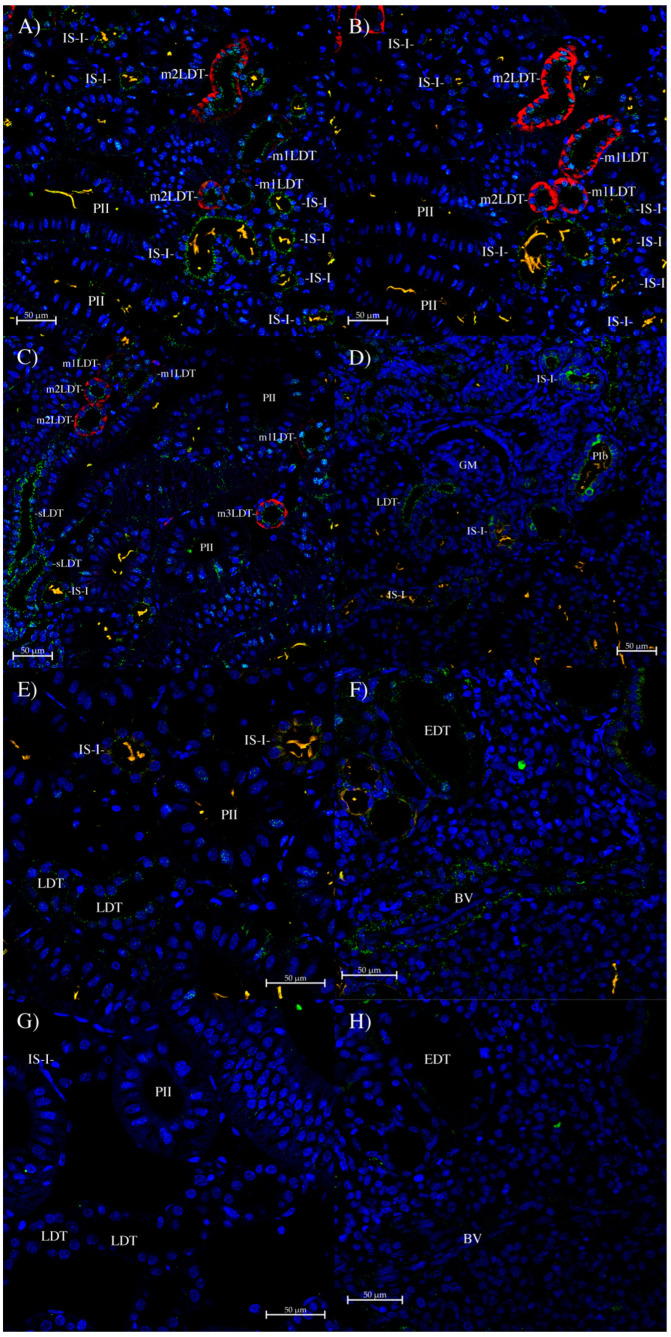
Immunohistochemistry using the UT-1/3 antibody. (**A**,**B**) Are serial sections stained with UT-1/3 (Green) and AQP3 (**A**) or AQP4/2 (**B**) antibodies (red). Sections show strong UT-1/3 antibody staining in the apical pole of IS-I tubules. The middle part of the LDT (m1 and m2LDT) also shows some apical pole staining in tubule cells. (**C**) AQP3 (red) shows graded staining along the LDT, with initially no staining (confirmed as LDT by AQP4/2 antibody). UT-1/3 staining at the start of the LDT (sLDT) was strong, with somewhat less UT-1/3 antibody staining in the middle LDT (which is m1, then m2, and then m3LDT). (**D**) Section showing UT-1/3 antibody staining in some PIb tubules. (**E**,**G**) are serial sections. (**E**) There was some IS-I and (weaker) LDT UT-1/3 antibody staining present on section (**E**). Section (**G**) was a control section incubated with peptide-antigen-blocked UT-1/3 antibody, and exhibited no similar staining (to that on section (**E**)). (**F**,**H**) are serial sections. (**F**) Shows very weak cytoplasmic UT-1/3 antibody staining in all five tubules of a bundle (denoted by EDT in the EDT tubule) in the bundle zone. A blood vessel (BV) also shows some UT-1/3 staining. Section (**H**) is a control section incubated with peptide-antigen-blocked UT-1/3 antibody and exhibits no similar staining (to that on section (**F**)). GM, glomerulus. On the sections, UT-1/3 antibody staining is green (Alexa 488 dye), anti-acetylated tubulin antibody cilia stain is orange (Alexa 555 dye), Squalus AQP3 and AQP4/2 antibody staining (see [12]) is red (CF633 dye), and Dapi nuclear counterstain is blue.

With the UT-1/3 antibody, the strongest staining was found in the IS-I tubule segment (Figure 8A), with strong staining also in the start of the LDT (sLDT, Figure 8C), which is denoted by no AQP3 staining (but with AQP4 staining). Moving along the LDT, the level of AQP3 staining increases from m1LDT through to m3LDT (this is still the middle of the LDT rather than the end due to still strong AQP4/2 staining; the end of the LDT has low AQP4 staining). There was little to no staining in the PII segment. In the bundle zone, there seemed to be a low level of generalized staining in all five bundle tubules (Figure 8F). There was some staining in a nearby blood vessel.

With all three of the UT-1 antibodies, staining was abolished by pre-incubation of the antibodies with their peptide-antigens (Figure 6F, Figure 7F, and Figure 8G,H).

## 4. Discussion

A study by Morgan et al. [23] in little skate showed that a urea uniporter protein like UT-1 was present in the apical (brush border) membranes of tubules in the bundle zone (dorsal kidney). This was in contrast to the staining seen here with the three UT-1 antibodies, which was also significantly different from that found in the studies by Hyodo et al. ([8]; in houndshark) or those of Imaseki et al. ([9]; in bullshark), who both found that UT-1 only showed significant expression in the bundle zone collecting tubule (CT) nephron segment. This was quite surprising and was the reason three antibodies were made to confirm these results. Although the results of the three dogfish-specific UT-1 antibodies here were not identical, none of them showed strong staining in any tubule that could be the CT in the convoluted bundle zone (such as in Figure 5A). In the straight bundle zone, the situation was a bit less clear as there are 2 distal tubules present, one with staining and one without. Due to the presence of UT-1 staining on either side of the IS-II straight bundle zone segment (IS-I and EDT in the convoluted bundle zone) and the lack of apparent staining in the part of the nephron immediately before the collecting tubule/CT (i.e., at the end of the LDT), this all suggests that the tubule with staining was the IS-II segment and the one without was the CT. Further ways to positively identify the different tubule segments in the bundle zone would be needed to be sure of this.

The different localizations within cells seen with the different antibodies suggest that there may be modifications to parts of the UT-1 protein, particularly in the case of the UT-1 and UT-1/3 antibody binding sites, that block the binding of these antibodies. The most likely candidates for this are phosphorylation of a serine or tyrosine in the binding site of UT1/3 and of a tyrosine in the binding site for the UT-1 antibody. It seems that these modifications may be required for the UT-1 protein to be moved from the ER/Golgi to subapical membrane vesicles and/or for movement to the apical plasma membrane. If this were true, it would explain why the UT-1 and UT-1/3 antibodies only bind cytoplasmic UT-1 protein, whereas (at least in IS-I) the UT-I/2 antibody seems to bind to UT-1 protein located in the apical membrane and would also suggest that the UT-I/2 antibody was not strong enough an antibody to detect cytoplasmic UT-1 proteins. Apical location of the UT-1 protein has been shown to be dependent on the external environmental salinity in the houndshark [24], which shows the membrane presence of the protein is regulated. Overall, the 3 antibodies concur that the strongest UT-1 staining was found in the IS-I and PIb nephron segments in the first sinus zone loop, closely followed by the start and middle of the LDT of the second sinus zone loop. This was to some extent in agreement with the study by Kakumura et al. [14], who, in the closely related holocephalon/chimaera elephant fish, showed that the IS-I had UT-1 expression (as well as the CT). Additionally, the sinus zone localization of UT-1 is supported by data suggesting that when sodium reabsorption was blocked by furosemide (blocking NKCC-2 in the EDT), “urea reabsorption was not diminished” and that “very large amounts of urea can be absorbed passively in the proximal tubule” [25]. UT-1 is known to be a passive facilitative urea transporter.

Another aspect of the study is the fact that UT-1 staining was seen to be much greater in some nephrons than others. This suggests that either the expression of UT-1 is patchy along the length of the nephron, for which there was some evidence, or that only some of the nephrons were operating. That has been shown before in studies on *Scyliorhinus caniculus* that showed that there is a blood vessel shunt that can bypass renal glomeruli and that some nephrons additionally were perfused but not filtering [26,27]. In line with this, another study on Stingrays acclimated to diluted SW concluded that they had “a remarkable (glomerular and tubular) functional reserve that is invoked to rapidly excrete water” [28]. If this were the case also in the dogfish, it would suggest animals would have an innate capacity to increase or decrease urine production depending on circumstances, but that any nephrons newly utilized would need to ramp up expression of proteins such as UT-1 for urea absorption.

The antibodies were also used to detect UT-1 proteins in Western blotting (Figure 4). With the UT-1 antibody, two protein bands were seen, which could correspond to UT-1 long and UT-1 short proteins (calculated size, 51.6 and 43.4 kDa, respectively). Both UT-1 short and UT-1 long have N-glycosylation sites (3 and 5, respectively), which may increase their sizes by an undetermined amount. The fact that the QPCR amplifications showed that the expression of UT-1 short was around 100× that of UT-1 long, suggests that the upper band may not be UT-1 long (as it might be too low to be seen). On the other hand, the levels of mRNA and protein abundance do not necessarily need to parallel each other. Translation of protein off a low abundance mRNA could potentially be much higher than that off a high abundance mRNA transcript. However, if the upper band really was not UT-1 long (maybe a different, larger glycosylated form of UT-1 short), it might explain why the UT-1/3 antibody did not detect much of that band. However, there was consistency between the antibodies detecting at least the 46 kDa band as well as a 52.5 kDa band (with UT-1 and UT-1/2 and, to a small extent, UT-1/3 antibodies). The fact that the UT-1/2 antibody failed to detect these protein bands in crude membrane extracts underlines how weak the binding of that antibody was. Outside of the 2 bands already mentioned, it is unclear what the identities of the other bands the UT-1/2 antibody detected are.

The quantitative PCR (QPCR) results underline with the RPL-P0 result that when something fundamental is performed on an animal, in all probability every gene may have its expression altered to some extent, and that undermines the case for the use of housekeeping genes in such circumstances. Moving the dogfish to different salinity environments seems to have produced a spur to the protein synthesis machinery to ramp up, at least in terms of RPL-P0 ribosomal protein mRNA being increased, significantly in 120% SW-acclimated dogfish (Figure 3A). Because these experiments generated relatively large amounts of total RNA (hundreds of micrograms), it is consequently easy to measure and normalize the RNA samples using agarose gel electrophoresis, using 18S rRNA levels. Ribosomal RNAs represent greater than 90% of total RNA and, therefore, are a proxy for the total amount being used. If only tiny amounts of total RNA were available, then it would be necessary to fall back on using housekeeping genes for gene expression normalization purposes. The expression of UT-1 long and short mRNAs was also both higher in 75% and 120% SW-acclimated animals, but only UT-1 long’s increase in 75% SW was significantly higher (Figure 4C). The 75% SW environment had a slightly greater effect on UT-1 long and short mRNA expression than the 120% SW environment did, whereas with Brain UT mRNA, it was the other way around, with a large increase only found in 120% SW-acclimated fish, although that was not statistically significant. In a similar way, results from expression studies previously with the same samples showed that AQP4 and AQP15 mRNA expression was significantly lower in 120% SW-acclimated dogfish, whereas AQP3 mRNA levels showed a similar expression profile to Brain UT [20,21]. The changes in UT-1 mRNA expression are most similar to those found in the Bullshark, where UT-1 mRNA expression was higher in lower (freshwater) salinity [9]. In houndshark, contrasting results were found. UT-1 mRNA expression was lower in 30% SW compared to 100% or 130% SW-acclimated fish [24]. With results in various species in opposing directions, it is hard to interpret what the significance of mRNA expression changes are, but as with the results here, it suggests that there may be some major differences in the mechanisms of renal urea reabsorption in different elasmobranch fish species.

The discovery of a second UT-1 gene (Brain UT) in dogfish [11] was of interest, and so the sequence of it was completed here, and a splice variant of it was also identified in the brain. However, the level of expression in the kidney was shown to be very low both in tissue PCRs (Figure 2) and in QPCR experiments (Figure 3), and so it may not be particularly relevant to renal function unless all the mRNA/protein for it shows a very limited and concentrated localization of expression. It was deemed not worth making an antibody against the protein, and cross-reactivity with the current three UT-1 antibodies is probably unlikely. However, as the tissue PCR shows, Brain UT is likely to have much more important functions within the dogfish brain and eye.

Lastly, the bundle zone has a potential role in reabsorption processes in general but also potentially for urea (in at least some elasmobranch species, in the CT) in particular. So what is the reason for the existence of the bundle zone with its surrounding peritubular bundle sheath that encloses it? A countercurrent mechanism for the bundle zone has been invoked due to the nephron loops within it [2]. Usually, the reason for having an enclosed area is to allow the concentration of something above levels normally present in body fluids. The most obvious possibility for that in the bundle zone would be that it allows the concentration of sodium ions. It is known that sodium ions are reabsorbed from forming urine in the bundle zone ‘diluting segment’, the EDT, via an NKCC2 cotransporter [12,14,29]. But every vertebrate cell has to have a certain level of Na,K-ATPase, usually on its basolateral membrane. This would put sodium ions into the extracellular fluid within the bundle zone. Evidence from Hentschel et al. [30] in skate kidney suggests that this is the case, as they found the sodium concentration was higher in the bundle zone than in the sinus zone. Additionally, the water permeability of the EDT and CT nephron segments (as well as possibly the other bundle zone nephron segments) has been suggested to be low [29], partly because there are no apically located aquaporin water channels that have been shown to be expressed in the bundle zone tubules to allow water out of the urine, although a paracellular pathway for water reabsorption cannot be ruled out. These facts would allow the concentration of sodium ions in bundle zone extracellular fluid, which would then enhance the sodium gradient across basolateral membranes of tubule cells that could be used for the augmented reabsorption of other solutes using sodium-solute exchangers. Whether those solutes would include urea would depend on the location and type of urea transporters. Although, as mentioned already, some elasmobranch species express apical UT-1 in the CT tubule, a basolateral exit pathway for urea into the bundle zone extracellular fluid has yet to be identified there. In order to get efficient reabsorption of urea at the level of 80–99% of that in the glomerular filtrate, active or secondary active transport of urea would be necessary, and there is some evidence for the existence of sodium-linked secondary active transport of urea in the dogfish [31]. Till date, any secondary active transporters of urea have yet to be determined. But it is possible to speculate that if they do exist in dogfish, they would most likely be found in the CT, which has been hypothesized to be a major site of urea absorption [29]. Then, you would have passive facilitative transport of urea (via UT-1) prior to the CT to remove much of the urea from the urine, followed by secondary active transport to remove the remainder that is reabsorbed in the CT. Some indirect evidence that suggests at least the majority of urea is absorbed in the sinus zone of the kidney comes from Hentschel et al. [30], who showed that the concentration of urea in skate kidney was lower in the bundle zone than in the sinus zone. Hence, a second-place secondary active urea transporter could be expressed in the sinus zone loops, especially the PII segment of the first loop. There is some evidence that the PII segment shows relatively high levels of urea reabsorption [32]. Although in the dogfish in places where apical UT-1 is expressed (PIb and IS-I), any secondary active transporters would be expected to be on the basolateral membranes of tubule cells. As with UT-1 proteins, in elasmobranch species, where various secondary active urea transporters would be located would likely be different across different elasmobranch taxonomic groups.

Of course, some water is needed to generate bundle zone extracellular fluid, and that seems to come through the peritubular bundle sheath [15], which expresses aquaporin water channel membrane proteins, [12]. Solutes reabsorbed in the bundle zone would attract water by osmosis. Added fluid would then produce hydrostatic pressure that would push the fluid out of the bundle zone (i.e., using a bulk flow mechanism) presumably into the sinus zone blood sinuses. In some elasmobranch species, there have been shown to be central vessels in bundle zone bundles to conduct fluid [33]. It is not clear in the dogfish whether central vessels are present or not; some bundles seem to have vessels (see Figure 5A as a possibility) and others not. Where central vessels exist, there are issues concerning how water and solutes would access the inside of the vessels from the extracellular fluid surrounding bundle tubules. So, there are many aspects of urea, water, and other solute transport/reabsorption in the kidney of elasmobranchs still to be addressed.

## Figures and Tables

**Table 1:** Primer sequences (fragment sizes generated in parenthesis where appropriate).

**PCR Amplification and RACE Primers**	
**Short and Long UT-1 Tissue PCR**	
Sense (Common)	ATCAGTGAGGACAGGAACGTCTTGA
Antisense (Short; 1363 bp)	AGGGACACATTTCATCTGTGGGA
Antisense (Long; 1518 bp)	GCCCTTAGACTATCCTGTCAGCAACA
**Brain UT Tissue PCR (499 bp)**	
Sense	CCGTCCAACAAGATAACGACAAGAATGTCTACTGT
Antisense	TGCGGCCATATACACCGACAGAGCA
**Brain UT 5′ End (718 bp)**	
Sense	CTCGGGCAAACATTCCGGCATTAACTACAGTCTG
Antisense	TGCGGCCATATACACCGACAGAGCA
**Brain UT 3′ RACE PCR**	
3′ Race 1	GTACAATGGCATCTTGGTTGGCCTTCAACT
Nested 3′ RACE 2	GGAGATTGGTATTGGTGGCTACTACTGCCT
**RPL-P0 (775 bp)**	
Sense	AGGGAAGACAGAGCTACGTGGAAGTCCA
Antisense	AATGGGAAGGAGTAGTCTGTCTCCACAGC
**QPCR primers**	
RPL-P0 Sense (126 bp)	GGAGAACAATTCTGCTTTGGAAAAGCTCCTGCCT
RPL-P0 Antisense	GCAGCAGCTGGGACCTTGTT
UT-1 Short Sense (427 bp)	GGTATACTGGCAGGATTATCCCT
UT-1 Short Antisense	GCGTTGCATGTATATATATCTTGTACACAGT
UT-1 Long Sense (245 bp)	ACTCATGAACATCATGTCTGTGGTCGGA
UT-1 Long Antisense	CATTACTTCTTGGTTGCGGCGTTGT
Brain UT Sense (260 bp)	GTGTCAATAGTGTGCCCTGTGCTT
Brain UT Antisense	GGATTGTCGCAGCCATACACCTG

## Data Availability

Data from the article can be provided to anyone upon request.
